# Merkel Cell Carcinoma: New Trends

**DOI:** 10.3390/cancers13071614

**Published:** 2021-03-31

**Authors:** Ellen M. Zwijnenburg, Satish F.K. Lubeek, Johanna E.M. Werner, Avital L. Amir, Willem L.J. Weijs, Robert P. Takes, Sjoert A.H. Pegge, Carla M.L. van Herpen, Gosse J. Adema, Johannes H. A. M. Kaanders

**Affiliations:** 1 Department of Radiation Oncology, Radboudumc, 6525 GA Nijmegen, The Netherlands; e.zwijnenburg@radboudumc.nl (E.M.Z.); gosse.adema@radboudumc.nl (G.J.A.); 2 Department of Dermatology, Radboudumc, 6525 GA Nijmegen, The Netherlands; satish.lubeek@radboudumc.nl; 3 Department of Surgery, Radboudumc, 6525 GA Nijmegen, The Netherlands; annelies.werner@radboudumc.nl; 4 Department of Pathology, Radboudumc, 6525 GA Nijmegen, The Netherlands; avital.amir@radboudumc.nl; 5 Department of Maxillofacial Surgery, Radboudumc 6525 GA Nijmegen, The Netherlands; willem.weijs@radboudumc.nl; 6 Department of Head and Neck Surgery, Radboudumc, 6525 GA Nijmegen, The Netherlands; robert.takes@radboudumc.nl; 7 Department of Radiology and Nuclear Medicine, Radboudumc, 6525 GA Nijmegen, The Netherlands; sjoert.pegge@radboudumc.nl; 8 Department of Medical Oncology, Radboudumc, 6525 GA Nijmegen, The Netherlands; carla.vanherpen@radboudumc.nl

**Keywords:** Merkel cell carcinoma, surgery, radiotherapy, immunotherapy, biomarkers

## Abstract

**Simple Summary:**

In this review, we discuss a rare skin cancer that occurs mostly in elderly people called “Merkel cell carcinoma” (MCC). The incidence is increasing due to ageing of the population, increased sun exposure, and the use of medication that inhibits the immune system. Unlike most other skin cancers, MCC grows rapidly and forms metastases easily. We discuss the biology and treatment of MCC. Management should be by an experienced and multidisciplinary team, and treatment must start quickly. The standard practice of MCC treatment is surgery followed by radiotherapy. However, because it concerns an elderly and often frail population, (extensive) surgery may not always be feasible due to the associated morbidity. In those situations, radiotherapy alone is a good alternative. An important new development is immunotherapy that can cause long-lasting responses in a significant proportion of the patients with recurrent or metastatic MCC.

**Abstract:**

Merkel cell carcinoma (MCC) is a rare neuroendocrine tumor of the skin mainly seen in the elderly. Its incidence is rising due to ageing of the population, increased sun exposure, and the use of immunosuppressive medication. Additionally, with the availability of specific immunohistochemical markers, MCC is easier to recognize. Typically, these tumors are rapidly progressive and behave aggressively, emphasizing the need for early detection and prompt diagnostic work-up and start of treatment. In this review, the tumor biology and immunology, current diagnostic and treatment modalities, as well as new and combined therapies for MCC, are discussed. MCC is a very immunogenic tumor which offers good prospects for immunotherapy. Given its rarity, the aggressiveness, and the frail patient population it concerns, MCC should be managed in close collaboration with an experienced multidisciplinary team.

## 1. Introduction

Merkel cell carcinoma (MCC) is a rare and aggressive malignancy of neuroendocrine origin that originates in the skin. MCC is mostly seen on the facial skin and extremities of elderly Caucasians and is associated with UV-exposure and infection with the Merkel cell polyomavirus (MCPyV). More than 70% of patients are above 70 years of age at diagnosis [1,2]. Clinically negative prognostic indicators for survival are tumor burden, regional metastases, gender (male), location in face or trunk as compared to upper extremities, and immunosuppression [1,2,3,4,5]. Histopathological negative prognostic factors are depth of invasion, lymph vascular invasion, and T-cell infiltration [6,7]. Association with MCPyV is reported in more than 80% of MCC patients in the Western world [8].

The clinical presentation of MCC is a fast growing, painless, reddish-purple cutaneous nodule. The incidence of MCC is low, but increasing in most Western countries [9]. In the US, there was a 95% increase in the absolute number of cases reported to SEER-18, from 334 in 2000 to 652 in 2013 [10]. In Australia, the incidence among men increases at an annual rate of 4.2%. Remarkably, while the incidence of MCC in men is rising, the incidence in women has been decreasing since 2002 [11]. The reason for this is unclear. Most likely causes for the overall increase are ageing of the population, increased UV-exposition, both voluntary (sun bathing) and involuntary (outdoor jobs, depletion of the ozone layer) [12], and increased use of immunosuppressive medication. Additionally, better recognition by pathologists with the availability of more specific immunohistochemical markers for this tumor may have contributed. Tumors that were previously classified as “unspecified small cell carcinoma” may now be identified as MCC.

Patients with MCC are at a high risk for loco-regional recurrence and distant metastases. Like melanoma, MCC shows a strong tendency to form satellite lesions in the skin. At presentation, 50–65% of patients have localized disease, 25–50% have regional metastases, and about 10% present with distant metastases [13,14]. The variation in reported incidence of lymph node metastases can be explained by variations in diagnostic procedures, i.e., use of ultrasound with or without fine needle cytology, FDG-PET-scan, and/or sentinel node procedure. The 5-year survival, independent of age is 50–60% for localized disease, and for lymphogenic and hematogenic metastatic disease this is 30–35% and 14%, respectively [1,13,14,15]. It must be noted that in this elderly population, a significant number of MCC patients die due to other causes. In a Dutch cohort of 351 patients, the 5-year overall survival was 58%, but the MCC-related survival was 78% [16]. Farley et al. reported a high overall 5-year survival rate of 70% and a 5-year disease-specific survival of 84% [17].

## 2. Etiology, Pathology, and Tumor Biology

On routine hematoxylin and eosin staining MCC is typically characterized as a monomorphous small round blue cell tumor with round or oval nuclei, finely dispersed chromatin, indistinct nucleoli, and scant cytoplasm. There is a high mitotic rate. Variations are seen, in particular in MCPyV-negative MCC. Histopathological confirmation of MCC requires immunohistochemistry to differentiate from other small cell neoplasms such as metastatic small-cell lung cancer, melanoma, or lymphoma. Molecular markers diagnostic for MCC include neuroendocrine markers (chromogranin A, synaptophysin, CD56), cytokeratin 20 (dot-like pattern), neurofilament, and MCPyV large T antigen (LT). Negative TTF1, S-100, and leukocyte common antigen (LCA) can be used to differentiate MCC from small-cell lung cancer, melanoma, and lymphoma, respectively.

The small round blue cells of MCC share ultrastructural and immunohistochemical characteristics with benign Merkel cells. However, the suggestion that the Merkel cell is the cell of origin of MCC is debated. Benign Merkel cells are typically located in the basal layer of the epidermis whereas MCC is reported to originate from any layer of the skin. Furthermore, it is suggested that MCPyV-positive and negative MCC may arise from distinct cells of origin [18]. Additionally, morphological and immunophenotypical features differ between MCPyV-positive and negative MCC [19,20]. Virus-negative tumors have more heterogeneous cytological features and frequently display elongated nuclei, resembling the spindle-shape variant of small cell lung cancer, larger cell size, more abundant cytoplasm, and prominent nuclei. There is evidence that UV-associated MCC derives from an epidermal progenitor cell, whereas MCPyV-associated tumors are suggested to be of non-epithelial origin or, alternatively, from cutaneous appendage precursor cells [21]. 

MCPyV seroprevalence in the general population is very high and increases with age from 50% in childhood to 80% in adults above 50 years of age [22,23]. MCPyV remains latent in most immunocompetent hosts, but when the immune system weakens, this can lead to viral reactivation. MCPyV is a small circular double-stranded DNA virus with a genome of about 5400 base pairs that is divided into early and late regions [24,25]. The early region encodes large and small tumor (T) antigens and a 57 KDa protein of uncertain function. The late gene region encodes structural proteins, the major capsid protein VP1, and the minor capsid proteins VP2 and VP3. Despite the high MCPyV seropositivity in the population, the incidence of MCC is very low. This is explained by increasing evidence for the hypothesis that oncogenesis by MCPyV requires the rare combination of two essential events. First, clonal integration of the viral genome in the host genome must take place. A crucial next event is a mutation with loss of expression of the C-terminus of the “large T antigen”, by which viral replication is inhibited with a subsequent increase in the synthesis of the viral oncoproteins large T antigen and small T antigen that promote cell cycle progression and survival [18]. MCPyV-positive MCC typically carry a low mutational load whereas UV-induced oncogenesis of virus-negative MCC is characterized by a cascade of oncogenic mutations as a result of accumulating DNA-damage [18].

There is equivocal data on the prognostic significance of MCPyV-status [18]. One retrospective analysis of 282 cases used large T antigen immunohistochemistry with two distinct antibodies as well as quantitative MCPyV PCR to assess MCPyV-status [26]. Fifty-three of 282 MCC (19%) were identified as virus-negative. In multi-variate analysis including stage, age, gender, and immune status, virus-negative patients were 1.5 times more likely to die from MCC albeit that this was not a statistically significant difference (*p* = 0.14). The favorable outcome of MCPyV-positive MCC is linked to increased immune-cell infiltration, in particular of CD8+ T cells, suggesting an antitumor immune response as the underlying mechanism [18,27].

Ongoing expression of viral oncoproteins is necessary for survival and progression of MCPyV-associated MCC [28]. These persistently expressed viral antigens may at some point trigger a host immune response [29,30]. Spontaneous regressions of MCC have been reported, either or not after discontinuation of immunosuppressive medication. In patients that do not use immunosuppressives, spontaneous regressions have been reported shortly after biopsy. Likely, the biopsy can generate an inflammatory environment stimulating antitumor immune responses [31,32,33,34].

## 3. Diagnostics and Staging

Physical examination includes assessment of the primary tumor and documentation by light photography, palpation of regional lymph node basins, and inspection of the entire skin surface by a dermatologist. The latter is important because MCC patients often develop other skin cancers as well and the dermatologist is the best qualified professional to do this. In case of advanced primary tumors a CT- or MR-scan can be considered to assess invasion of deeper structures. There is no general consensus on the role of imaging in the work-up of MCC patients with clinically localized disease and the current NCCN practice guideline does not recommend routine baseline imaging [35]. However, a recent retrospective analysis revealed that of 492 patients with no signs or symptoms of regional or distant spread, 65 (13%) were upstaged by diagnostic imaging (CT, MRI, or FDG-PET-CT) with consequences for treatment [36]. Confirmative data are provided by a literature review on the role of FDG-PET-CT [37]. In addition, or as an alternative to PET-CT, ultrasound with fine needle cytology can be considered for the evaluation of regional lymph nodes [38,39].

However, guidelines and reviews concur that the preferred diagnostic method for assessment of lymph node status is sentinel lymph node biopsy (SLNB) [18,35,37,38,39,40]. In 25–45% of patients clinically staged N0, SLNB demonstrated lymph node metastases with the majority of studies indicating a number close to 30% [41,42,43,44,45,46,47,48,49]. Various predictors for sentinel lymph node positivity have been identified including tumor size, but even for tumors <1 cm and <0.5 cm, the risk is still 20–31% [42,46,47] and 14% [44], respectively. Based on clinical and pathological characteristics, no subgroup of patients could be identified to have a likelihood of a positive sentinel lymph node lower than 15% [42]. An analysis of 1174 patients undergoing SLNB yielded a hazard ratio of death of 3.15 (95% CI 1.98–5.04, *p* < 0.001) for patients with positive sentinel lymph nodes versus those with negative sentinel lymph nodes [46]. These data strongly support the recommendation of SLNB for assessment of regional lymph nodes in MCC. However, in none of the patient cohorts discussed above, PET-CT or ultrasound with or without cytology was routinely performed in the diagnostic work-up. What the added value of SLNB is after state-of-the-art imaging is an issue that needs to be further addressed. Of patients with clinically uninvolved regional nodes 17% were upstaged by PET-CT, because of detection of regional and/or distant metastases, indicating that futile SLNB can be avoided in these patients [36,37].

Staging is according to AJCC, 8th edition which is based on an analysis of prognostic factors from 9387 MCC cases in the US [13].

## 4. Treatment

For various diseases with high prevalence at old age such as rheumatoid arthritis, psoriasis, and chronic lymphatic leukemia, immunosuppressive medication is frequently prescribed. It has been suggested that patients affected by rheumatologic diseases and treated with biologic immunosuppressives, including anti-TNF, are at an increased risk of MCC development [50]. Due to this possible cause-effect relationship, after diagnosis of MCC, immediate discontinuation of this medication should be considered, at least temporarily [5]. If discontinuation is not possible, dose reduction or replacement medication with less immunosuppressive effect may be an alternative. Burden and risk of progression of the underlying disease must be weighed against the potential detrimental effects on the tumor. For solid-organ transplant recipients, this is a particular problem because there is the risk of losing the transplanted organ. It is not known if restart of immunosuppressives after an adequate disease-free interval is safe or if drugs with another mechanism can be an alternative option.

### 4.1. Surgery and Adjuvant Radiotherapy

Radical excision is generally considered the treatment of choice [35,38,39]. Wide resection margins, varying between 1 cm and 3 cm depending on localization, are recommended. The reason for this is not only to obtain free resection margins, but also to include potential small satellite lesions close to the primary tumor. Wide excision must be balanced against the functional and cosmetic consequences, especially for tumors arising in the facial skin. The recommendation for wide excision is mostly based on experience from the 20th century when patients were often treated with surgery alone and local recurrence rates were in the order of 25% to 45% [51,52,53]. A more recent retrospective analysis of a cohort of 240 patients of whom 70% received postoperative radiotherapy reported much lower local recurrence rates of 2.9%, 2.8%, and 5.2% for margins of 1 cm, 1–2 cm, and >2 cm, respectively [54]. This suggests that if postoperative radiotherapy is given routinely, margins of 1 cm should be sufficient. More recent studies demonstrate that patients with localized disease that undergo surgery and postoperative radiotherapy not only have a significantly better loco-regional tumor control, but also a better survival compared to those treated with surgery alone. Data were extracted from two US databases: SEER database (National Cancer Institute) and National Cancer Database (American College of Surgeons). Large cohorts of 1665, 4815, and 6908 cases were analyzed for the role of adjuvant radiotherapy in MCC [15,55,56]. Adjuvant radiotherapy improved 5-year overall survival rates by 10–15% depending on the size and stage of the tumor. Even for small primary tumors, there was a survival advantage [55]. A recent meta-analysis (29 studies, 17,179 patients) confirmed that adjuvant radiotherapy improves survival significantly (HR 0.81, *p* < 0.001) and reduces the risk of local and regional recurrence by 80% and 70%, respectively [57]. Apart from the fact that MCC is very radiosensitive, with radiotherapy, much larger skin surfaces and wider lymph drainage areas can be treated than with surgery. Whether small node-negative tumors should be routinely treated with postoperative radiotherapy remains a matter of debate. Even in a subgroup analysis of 168 patients with tumors <1 cm, Mojica et al. reported an improved median survival from 48 to 93 months with adjuvant radiotherapy [55]. Frohm et al., on the other hand, reported acceptable loco-regional control rates with surgery alone in tumors with largest diameter <2 cm [58]. Of 104 patients, 18 (17%) developed a local, in-transit, and/or regional recurrence. Two comments are to be placed with this study: First, the 17% is an absolute rate, the actuarial rate (corrected for duration of follow-up) most likely is higher, and second, the majority (13) of the recurrences were in the regional lymph nodes, which is a poor prognostic sign. Based on these and other data, the Danish guideline suggests that adjuvant radiotherapy may be omitted in selected low-risk cases, i.e., primary tumor <1 cm, negative margin status, no lymph vascular invasion, negative sentinel node biopsy, and no chronic immunosuppression [36].

After a negative SLNB, the risk of regional recurrence is relatively low (9–16%) [40,41,45] and treatment of the lymph node regions is generally not recommended. A positive SLNB must be followed by a complete lymph node dissection or regional radiotherapy. Despite subsequent treatment, the cumulative regional recurrence rate in SLNB positive patients is 11–28% [43,48]. If no SLNB is performed, elective treatment of at least the first draining lymph node level either by surgery or by radiotherapy is recommended. In case of clinically manifest regional metastases, a therapeutic lymph node dissection is performed. In virtually all cases, postoperative radiotherapy is indicated because it reduces the regional recurrence risk and improves the 3-year disease-specific survival from 48% to 76% [59].

The target volume for radiotherapy includes the primary tumor bed after excision with a margin for microscopic spread. The Danish guideline recommends margins of 1–2 cm, but this is not evidence-based [39]. Expert opinion is that margins should be generous, up to 3 cm, but adjusted to critical structures and sensitive organs, especially in the face. In case of clinically node negative disease, the regional nodal stations should be irradiated electively. This is not indicated after a negative sentinel node procedure. If lymph node metastases are present, the positive nodal level is treated as well as the next draining level. 

A dose of 50–56 Gy in 2-Gy fractions is recommended in case of negative resection margins, 56–60 Gy for microscopically positive margins, and 60–66 Gy for grossly positive resection margins [35]. Depending on the condition of the patient, the size of the target volume and vulnerability of the tissues to be irradiated, hypofractionated schedules with biologically equivalent tumor dose can be used.

Side effects during and shortly after radiotherapy include dry or moist desquamation of skin. Other acute side effects depend on the area treated. Treatment of regional nodes in the head and neck area can cause mucositis with dysphagia. Skin and mucosal reactions generally heal within a few weeks. Hair loss, fibrosis, lymphedema, and xerostomia are potential long-term effects. These are generally mild with modern radiation techniques such as intensity modulated radiotherapy (IMRT) and volumetric modulated arc therapy (VMAT). The risk and severity of lymphedema increase if postoperative radiotherapy is applied after lymph node dissection [60]. 

### 4.2. Definitive Radiotherapy

MCC is notorious for its rapid growth and metastatic potential. After excision, re-excision, or lymph node dissection, it is not unusual that there is a delay before adjuvant radiotherapy is started [61]. The reason for this is multifactorial. Patients are mostly elderly and frail and have multiple comorbidities. Prolonged postoperative recovery and wound healing disturbances are common. Furthermore, because of its rarity, unfamiliarity with the disease still often causes unwanted delays in referrals and treatment. Two recent studies analyzed the time elapsed between surgery and radiotherapy, and concurred that the risk of loco-regional recurrence increased if the delay was greater than 8 weeks (25% vs. 10% and 37% vs. 0%, *p* < 0.01) [62,63]. These findings are supported by an earlier Australian publication [64].

**Figure 1:**
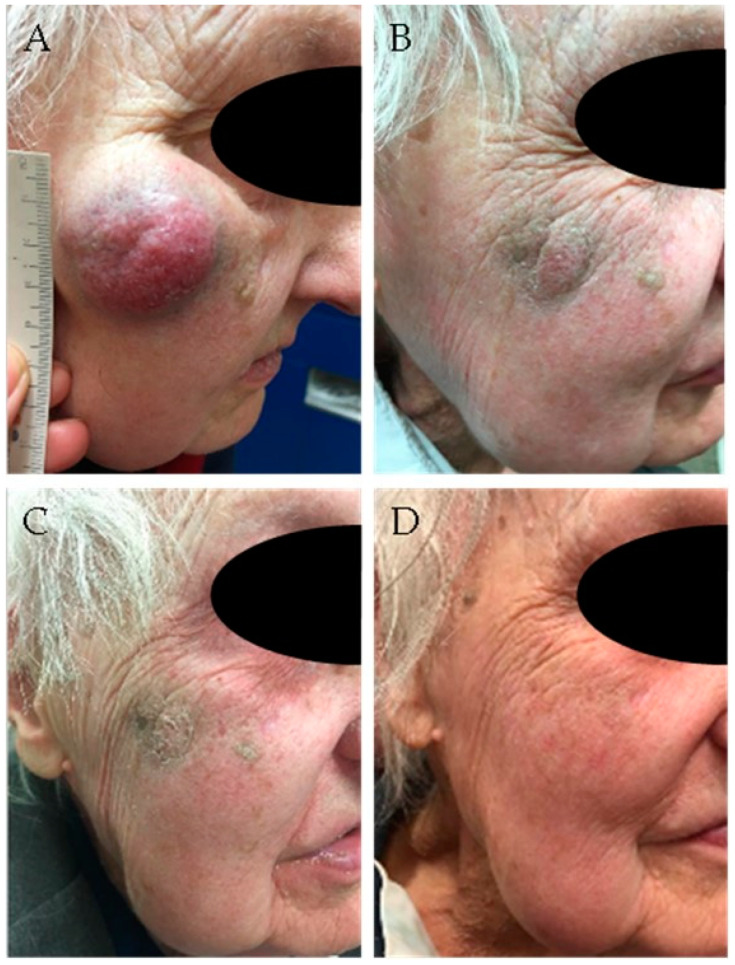
Ninety-four-year-old lady with Merkel cell carcinoma (MCC) of the right cheek, treated with radiotherapy (48 Gy). (**A**) Before treatment; (**B**) after 8 fractions (32 Gy); (**C**) after 12 fractions (48 Gy); (**D**) 6 weeks after completion of radiotherapy.

Definitive radiation monotherapy is used as an alternative to surgery for patients who are poor surgical candidates or for those in whom surgery would result in significant functional compromise [65]. Given this selection bias, it is difficult to compare the results of primary radiotherapy versus surgery in retrospective cohorts. An attempt was made by a propensity score matched analysis using patient data from the National Cancer Database [66]. MCC patients treated with definitive radiotherapy were identified and matched with another patient treated with surgery (with or without adjuvant radiotherapy) accounting for age, co-morbidity score, stage, and grade. There were 1227 patients treated between 2004 and 2014 in each group. For stage I-II disease, 5-year overall survival was 61% in the surgery group and 42% in the radiotherapy group. For stage III, this was 34% and 21%, respectively. However, it is noteworthy that, despite the matching, there were significant differences between the groups. Patients in the radiotherapy group had larger and more advanced tumors, were less often treated in high volume academic centers, and had longer delays from diagnosis to start of treatment. The authors acknowledge these limitations and appreciate that “long-term survival can be obtained in patients with locally advanced and regionally metastatic disease with definitive radiotherapy”. Single center studies report good loco-regional control rates with radiotherapy alone, ranging from 75% to 95%, similar to results of single center studies with surgery plus adjuvant radiotherapy [16,59,67,68,69,70,71,72,73]. The studies with radiotherapy alone had smaller patient numbers, but also included more advanced stages. A systematic review including 23 studies encompassing 264 patients reported a cumulative post-radiotherapy in-field control rate of 88% [74]. Figure 1 shows a patient with a large MCC on the cheek that was treated with radiotherapy alone. There was a durable complete regression until the last follow-up one-and-a-half years later.

Although surgery is generally considered the primary treatment for MCC, this has developed empirically. There are no prospective clinical studies comparing surgery with or without adjuvant radiotherapy with radiotherapy alone. MCC is very radiosensitive. In vitro data confirm that it is even more sensitive than small-cell lung cancer, a tumor where radiotherapy and chemotherapy are the main treatment modalities and surgery is of minor relevance [75]. Therefore, primary radiotherapy is an excellent alternative for surgery with postoperative radiotherapy, also for operable patients with resectable disease [76]. A single modality treatment can spare these elderly patients the burden of additional morbidity and reduce health care costs.

### 4.3. Systemic Treatment

There is no role for chemotherapy in the primary treatment of MCC. Adjuvant chemotherapy for patients with stage I-III disease does not improve survival [15,35,77]. In the palliative setting, chemotherapy can be given for metastatic disease. Cytostatic drugs mostly used are carboplatin (or cisplatin) and etoposide or a combination of cyclophosphamide, doxorubicine (or epirubicine), and vincristine. There is often a rapid response (53–76%), but rarely is this long-lasting [77]. Additionally, these drugs are toxic for the elderly patient and often not tolerated. The progression-free survival varies from 3 to 8 months [77].

Chemotherapy will fade further into the background with the upsurge of immunotherapy. MCC is a very immunogenic tumor, indicating that there is great potential for immunotherapy. Avelumab is a fully human monoclonal antibody that targets the programmed death-ligand 1 (PD-L1). Expression of programmed cell death protein 1 (PD-1) ligands, in particular PD-L1, is upregulated in a variety of tumors including MCC, and blockade of PD-L1 signal can sensitize tumors to cytotoxic T lymphocyte killing [78]. In a phase II study that included 88 patients with metastatic MCC previously treated with chemotherapy, the immune checkpoint inhibitor avelumab produced a response in 33% of patients of which 11% had a complete response [79]. The median time to response was 6.1 weeks and was not associated with MCPyV or PD-L1 status. In 71% of responders, there was a durable effect of more than one year. Experience from daily practice showed higher response rates of 47–57% and complete response in almost 25% of the patients, albeit that the duration of response was shorter (median 8 months) [80,81].

Immunotherapy with the PD-1 inhibitor pembrolizumab as first-line treatment for irresectable recurrence or metastatic disease was studied in a cohort of 50 patients [82]. A complete response was observed in 24% of patients and a partial response in 32%. Additionally, in this study, responses were prolonged with a 2-year progression-free survival of 48%.

Recently, a study was published on nivolumab, another PD-1 inhibitor, in the neoadjuvant setting [83]. Patients with resectable MCC received one or two courses of nivolumab, once per two weeks, starting 4 weeks before tumor resection. Of 39 included patients, 36 were operated, and in 17 (47%) tumors, a pathological complete response was observed. Three patients did not undergo surgery because of tumor progression or side effects of the treatment. Four other patients had progressive disease under nivolumab. The observation that 7 of 39 patients (18%) experienced detrimental effects under the neoadjuvant treatment is not trivial. It means that in these cases, while the tumor is progressing, valuable time is lost with potential deleterious postponement of local treatment. This may adversely affect the prognosis and shows that it is important that patients are closely observed during neoadjuvant treatment. Additionally, this emphasizes the importance of developing biomarkers predictive for response to immunotherapy.

Avelumab is currently considered first-line treatment for metastatic MCC, but it is expected that soon immunotherapy will also take a role in the primary treatment of localized disease, either in the (neo)adjuvant setting or concurrently with local treatment, be it surgery or radiotherapy. Currently, 9 clinical trials that study the role of immunotherapy specifically in MCC are registered in clinicaltrials.gov (Table 1). Five of these are trials that combine immunotherapy with concurrent radiotherapy. The hypothesis is that the immune activating properties of radiotherapy can potentiate immunotherapy.

**Figure 2:**
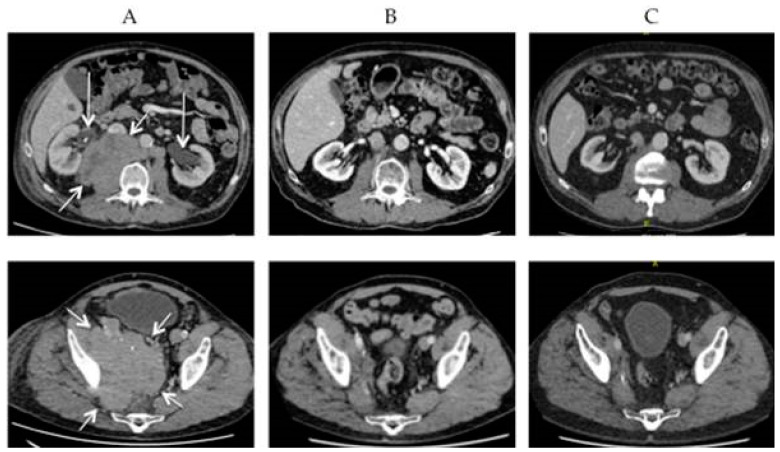
CT-scans of a 66-year-old man with massive lymphogenic metastases in abdomen and pelvis (short arrows) progressive under avelumab. Subsequent treatment with radiotherapy and restart of avelumab in last week of radiotherapy. (**A**) Before radiotherapy, note bilateral hydronephrosis (long arrows); (**B**) two months after radiotherapy (13 × 3 Gy), complete regression and recovery of hydronephrosis; (**C**) two years after radiotherapy; persistent complete regression.

Figure 2 shows a patient with extensive lymphogenic metastases from a MCC progressive under avelumab. Avelumab was discontinued, and palliative radiotherapy was initiated. In the last week of radiotherapy, avelumab was restarted. After two months, there was a complete remission and the patient is still free of disease at last follow-up two years later. Of interest in this context is that out-of-field abscopal effects have been reported following short-course radiotherapy in patients with MCC progressive on PD-1 checkpoint blockade [84]. These observations suggest that the combination of radiotherapy and immunotherapy may be a potent therapeutic strategy, not only for advanced metastatic disease, but also for earlier stages. Better understanding of the mechanisms behind the interactions between the two modalities will be obtained by current and future research [85].

Although immunotherapy is often better tolerated than many chemotherapy regimens, it is not without side-effects. The list of potential side-effects is long, but many are rare. The toxicity profiles of avelumab and pembrolizumab largely overlap and most frequent are infusion-related (allergic) reactions, fatigue, diarrhea, nausea, and fever. Toxicities more specific for immunotherapy include autoimmune endocrine dysfunctions, pneumonitis, colitis, and hepatitis [86,87]. Reported overall incidence of adverse effects for avelumab was 28–46%, of which 8–9% were grade 3–4 adverse reactions [80,83]. In the study with pembrolizumab as first line for advanced and metastatic MCC, treatment-related adverse events of any grade occurred in 48 of 50 (96%) patients, of which 14 (28%) were grade 3 or higher. Seven patients discontinued pembrolizumab as a result of treatment toxicity [82]. 

A strategy to avoid adverse events is intratumoral immunotherapy for accessible lesions. In a pilot study, 15 MCC patients were subjected to intratumoral delivery of plasmid interleukin-12 via electroporation [88]. All patients completed at least one cycle without noteworthy systemic toxicity.

For patients that do not respond to immunotherapy, alternative targeted therapies are needed. The mutational profile of MCPyV-positive tumors is different from that of MCV-negative tumors. It may be important to consider this biologic distinction when selecting a targeted therapy because driver mutations are more likely to be present in the MCPyV negative tumors that have a high mutational burden. About 80% of MCC’s are driven by integration of MCPyV and ongoing T-antigen oncoprotein expression is needed for tumor progression. T-cell responses to these antigens are reported [89], which might explain the rare cases of spontaneous regression [33,90] but in the vast majority of cases, this immune response is suboptimal and ineffective for various reasons. It has been suggested to enhance this immune response by therapeutic vaccination to T-antigen under the assumption that the “nonself” viral antigen can trigger a stronger and more tumor-specific response compared to less cancer specific overexpressed oncoproteins [91,92]. Therapeutic MCPyV vaccination has been explored in a murine melanoma tumor line [93]. It could be demonstrated that a vaccine encoding the amino terminus of MCPyV large T antigen generated an antitumor effect mediated by CD4+ T-cells. Another group exploited dendritic cells loaded with large T-antigen and showed induction of antigen-specific T-cell responses in blood from healthy donors and MCC patients [94]. MCC has been shown to be an excellent model for further exploration of therapeutic anti-tumor vaccination.

One recent mechanistic insight relates to the activation of lysine-specific histone demethylase 1 (LSD1)-mediated dysregulation of gene expression by MCPyV small T antigen [95,96]. It was observed that all of six tested MCPyV-positive MCC cell lines responded to LSD1 inhibition, whereas three MCPyV-negative MCC cell lines did not [95]. Another study showed that LSD1 is a potent inhibitor of anti-tumor immunity and responsiveness to immunotherapy [97]. This suggests that a combination of LSD1 depletion and PLD1 blockade is a strategy that needs to be explored in clinical trials.

A drug currently under clinical investigation in MCC is called domatinostat (4SC-202). Domatinostat inhibits both class I histone deacetylases (HDAC’s) and LSD1, and enhances the expression of major histocompatibility (MHC) class I and -II genes [98,99]. As a result, the immunogenicity of tumor cells is increased with improved recognition by cytotoxic T-cells. The combination with a checkpoint inhibitor is expected to improve the effect of immunotherapy, especially in patients that do not respond to anti-PD-(L)1 treatment alone. A clinical study combining domatinostat and avelumab in patients with advanced or metastatic MCC that have progressed on previous avelumab or pembrolizumab monotherapy is currently recruiting (NCT04393753). 

Other therapeutic opportunities include somatostatin analogues, tyrosine kinase inhibitors, electrochemotherapy, talimogene laherparepvec (TVEC), and many others. However, most of these strategies have not been investigated systematically in properly designed prospective clinical trials. Reports are mostly retrospective and involve small patient numbers.

## 5. Biomarkers

Biomarkers can be prognostic or predictive. “Predictive assays” need to be distinguished conceptually from “prognostic factors”. The latter are determined empirically and, although useful, they merely indicate favorable or unfavorable outcomes, but offer no basis for selection of more effective treatment strategies. A predictive assay provides a mechanistic basis and identifies a biological target for personalized treatment.

As prognostic molecular biomarkers for MCC, p63, p53, survivin, CD34, hedgehog proteins, and several others have been suggested, but none of these emerge as very strong and robust prognosticators [18]. The prognostic relevance of MCPyV remains equivocal [100]. Serum neuron-specific enolase (NSE) has become of interest recently. NSE is found in neuroendocrine tissues and is expressed in the cytoplasm of MCC cells [101]. NSE was determined in serum samples of 84 MCC patients at baseline and during follow-up [102]. Baseline NSE levels correlated with extent of disease, but not with relapse-free survival or overall survival. Interestingly, NSE was particularly useful in detecting progression of the disease with a negative predictive value of 98%. Another, earlier study in 60 patients, however, did not find associations of NSE blood levels with recurrence or survival [103]. The clinical relevance of NSE as a prognostic biomarker needs to be further validated.

Immune-response related tumor characteristics are the most likely predictive biomarker candidates for immunotherapy. In the three previously discussed phase II trials with immunotherapy MCPyV-status, total mutational burden (TMB), CD8+ T-cell density, and PD-L1 expression have been investigated as putative biomarkers [79,82,83]. For TMB and CD8+ T-cell density, non-significant trends for associations with tumor response were found. PD-L1 expression (≥ 1% tumor cells positive) varied from 26% to 82% in the three studies, and no associations were found with response to immune checkpoint blockade. In two studies, however, there was a trend for better overall survival for cases with positive PD-L1 expression [79,82]. The large variation in PD-L1 expression between the studies is remarkable. This might be explained by tissue sampling errors, differences in immunohistochemistry protocols, inter-observer differences, and/or differences between untreated and recurrent cases. In a retrospective analysis of a small cohort of 27 MCC patients, PD-1, but not PD-L1, expression was associated with immunotherapy response [104]. Response rate was 77% in PD-1 positive tumors vs. 21% in PD-1 negative tumors (*p* <0.01). The value of PD-L1 and PD-1 as predictive biomarkers for immunotherapy in MCC needs to be further explored in larger clinical trials.

## 6. Multi-Disciplinary and Expertise

Given the rarity and the aggressiveness of MCC and the rapid evolution of new treatment opportunities, management of MCC requires a multidisciplinary team, preferably in a high-volume center. An analysis of 5304 cases from the US National Cancer Database with stage I-III MCC demonstrated that 5-year overall survival was 62.3% at high volume facilities vs. 56.8% at lower-volume facilities (*p* < 0.001) [105]. That being said, it will not always be feasible or desirable to refer these elderly and frail patients to centers located further away. A solution can be to set up multidisciplinary consultation networks to provide the best attainable care for these patients near their own living environment.

## 7. Conclusions

MCC is a rare skin cancer, albeit with rapidly increasing incidence. Management requires a multidisciplinary team, preferably within a network of an expertise center. Tumor progression and metastasis formation is often fast, and early recognition of MCC and expeditious diagnostic workup and treatment initiation are vital. Effective treatments are available that have improved prognosis significantly. Surgery with adjuvant radiotherapy is the standard for localized disease, but radiotherapy alone is a good alternative. Immune checkpoint inhibitors offer durable responses in a significant proportion of the patients with metastatic or recurrent disease. However, treatment morbidity is not negligible in this elderly and frail patient population. The challenge is to accomplish as high as possible cure rates with limited and acceptable toxicity and morbidity. 

## Figures and Tables

**Table 1:** Clinical trials registered in ClinicalTrials.gov that study the role of immunotherapy specifically in MCC (date of search: 9 March 2021).

ClinicalTrials. Gov Identifier	Type of Study	Investigational Drug	Mode of Action	Eligibility	Recruitment Status
NCT02584829	Phase I/II	Avelumab *	PD-L1 inhibition	Stage IV	active, not recruiting
NCT04160065	Phase I	IFx-Hu2.0 (intratumoral)	Emm55 protein expression	Advanced	recruiting
NCT04291885	Phase II, randomized	Avelumab	PD-L1 inhibition	Stage I-III	recruiting
NCT03271372	Phase III, randomized	Avelumab	PD-L1 inhibition	Stage III	recruiting
NCT03988647	Phase II	Pembrolizumab *	PD-1 inhibition	Stage IV	recruiting
NCT03798639	Phase I, randomized	Nivolumab * ipilimumab	PD-1 inhibition CTLA-4 inhibition	pathol. Stage IIIA-B	recruiting
NCT03712605	Phase III, randomized	Pembrolizumab	PD-1 inhibition	Stage I-III	recruiting
NCT03304639	Phase II, randomized	Pembrolizumab *	PD-1 inhibition	Stage III-IV	active, not recruiting
NCT04261855	Phase Ib/II	Avelumab *	PD-L1 inhibition	Stage IV	recruiting

* Trials that combine immunotherapy with concurrent radiotherapy.

## Data Availability

Not applicable.
